# Effects of indoor plants on office workers: a field study in multiple Dutch organizations

**DOI:** 10.3389/fpsyg.2023.1196106

**Published:** 2023-06-28

**Authors:** Sjerp de Vries, Tia Hermans, Fransje Langers

**Affiliations:** ^1^Wageningen Environmental Research (WENR), Wageningen University & Research, Wageningen, Netherlands; ^2^Cultural Geography Group (GEO), Wageningen University & Research, Wageningen, Netherlands

**Keywords:** plants, office workers, workspace, well-being, functioning, indoor climate

## Abstract

In the period 2019–2020, the effect of plants in the workspace on (a) the physical indoor climate, (b) the perception of the workspace by office workers, and (c) their health, well-being and functioning was investigated in nine organizations. This paper reports the outcomes of the latter part. A conceptual model describing the short-term, medium and long-term effect of plants on people was formulated, containing 18 outcome variables. A “Before After Control Impact” quasi-experimental research design was used. A control workspace and an intervention workspace were selected in each of the organizations. A pre-measurement was conducted in both. Correlational analyses, based on the pre-measurements in all organizations and workspaces, confirmed the associations proposed by the conceptual model to a large extent. After placing plants in the intervention workspace, a maximum of two post-intervention measurements were conducted (due to COVID-19 not in all nine organizations), the last one at least 4 months after the introduction of the plants. Overall significant effects were found on complaints about dry air (fewer), the sense of privacy (higher), the attractiveness of the workspace (higher), satisfaction with the workspace (greater) and having a health-related complaint, especially when at work (fewer). The first three effects were already observed in the analyses only including the first post-measurement. The latter two effects only showed up in the analyses including two post-measurements. No direct effect of the plants could be demonstrated on the 13 other outcome variables. The observed effects mainly concern outcome variables that are positioned at the beginning of the proposed causal chain, starting with plants and ending with mental health, absenteeism and job satisfaction.

## 1. Introduction

The presence of vegetation in the residential environment has been shown to be associated with human health and well-being, physically as well as mentally (Houlden et al., 2018; De La Fuente et al., 2021). This is likely to be the case for cognitive functioning as well (Ricciardi et al., 2022). Experimental studies provide evidence of causality, at least for short-term effects, with regard to stress and cognitive functioning (Ohly et al., 2016; Kondo et al., 2018; Stevenson et al., 2018; Mygind et al., 2021). A usual assumption is that more frequent exposure to, or contact with the vegetation (visual or otherwise), will lead to an accumulation of such short-term effects and produce more structural benefits, in that way explaining the associations observed in large-scale cross-sectional studies. Most of the aforementioned research focused on outdoor vegetation. Less research has been conducted on the long-term effects of indoor plants, especially in real-life settings (Han and Ruan, 2019). However, based on the results for outdoor vegetation, indoor plants are also likely to contribute to human well-being and functioning, especially in places where people spend much of their time. Office workers spend a considerable amount of time in their offices.

Research on indoor plants in office settings is indeed suggestive of several effects, directly or indirectly contributing to the well-being of the employees. To start with, plants may increase the relative humidity in the office (Han and Ruan, 2020). Especially in winter time, the indoor air can become dry to the extent that it may results in complaints. Through evaporation, plants may increase the relative humidity. Plants may also improve the air quality by reducing the concentration of volatile organic compounds (VOCs, *ibid.*). Their capacity to do so is correlated with that of moisturizing the air. Plants may also help to reduce excessive CO_2_-levels in the air. Besides their effects on air quality, plant may help to reduce noise, although this capacity seems limited (Oseland and Hodsman, 2018). But apart from an effect on the (objective) noise level, plants may also have an effect on the experienced noise annoyance. The same objective noise level has been shown to result in less noise annoyance if plants are present (Mediastika and Binarti, 2013). A similar type of effect has been reported to occur with regard to room temperature. The range of temperatures that is perceived as acceptable is wider when plants are present in the office (Mangone et al., 2014). This implies that fewer complaints about the temperature being too high or too low may occur. Depending on their positioning, plants may also help to screen (oneself from) other occupants of the office, thereby increasing the sense of privacy. A lack of privacy is one of the complaints frequently associated with open plan offices (Kim and De Dear, 2013). Finally, the presence of (healthy) plants is also likely to make the appearance of the office more attractive (Evensen et al., 2017).

Either directly, or by way of the aforementioned effects, the plants may also contribute to a higher satisfaction with the workspace (Nieuwenhuis et al., 2014), a more positive/less negative mood (Han and Ruan, 2019), less perceived stress (Toyoda et al., 2020), being better able to concentrate (Nieuwenhuis et al., 2014), and fewer health-related complaints when at work (such as dry eyes, sore throat, headache; Evensen et al., 2013). Given that the beneficial mood and stress effects do not only pertain to oneself, but also one’s co-workers, the social climate, i.e., the way colleagues interact with each other, is also likely to be affected positively (Thomsen et al., 2011). Altogether, this makes it likely that one perceives one’s work as more pleasurable. Also one’s functioning is likely to improve (Bakker and Van Der Voordt, 2010), e.g., by being better able to concentrate by reduced noise annoyance (Banbury and Berry, 2005). Finally, the more pleasant, less stressful working environment are likely to reduce the need for recovery after that working day (Kraaijeveld et al., 2014). Also one’s job satisfaction is likely to be higher. And, because of effects such as a lower need for recovery, so is one’s mental health (not limited to work environment; Nieuwenhuijsen et al., 2016). Such effects, in turn, are likely to result in lower sick leave numbers (see, e.g., Soriano et al., 2018).

**Figure 1:**
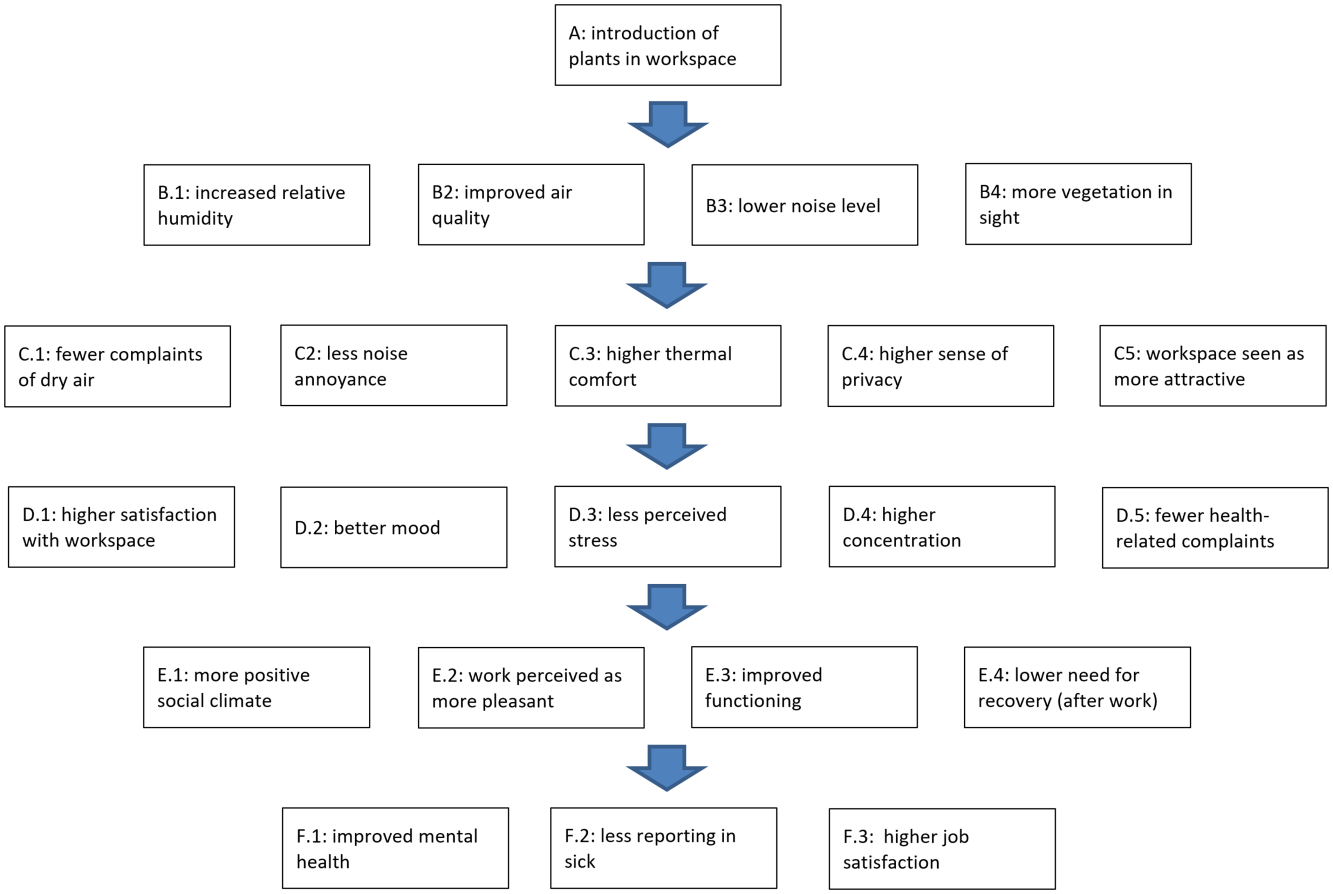
Conceptual model for effects of introducing plants in the workplace, roughly ordered from proximal to more distal effects. Reinforcing interrelations between effects are assumed to exist (not shown in figure). Most effects pertain to the workplace context (except E.4 and F.1–3).

In Figure 1, the above is summarized in a conceptual model of the hypothesized (beneficial) effects, roughly ordered from proximal to more distal effects. The model does not present all the positive interlinkages between the different effects, which are also likely (i) between effects at the same “level” and (ii) also from more distal to more proximal effects, once the latter have manifested themselves. However, it should also be noted that the more distal effects are expected to have weaker (direct) links with the introduction of the plants, because of increasing number of other, potentially more important determinants (not in model). For example, in our conceptual model we make a distinction between how pleasurable the employee finds it to perform his/her daily activities and his/her job satisfaction. The latter has been shown to be strongly associated with pay satisfaction (Cantarelli et al., 2016). We assume that this is the less the case for daily work pleasure, and, conversely, that work pleasure is likely to be stronger influenced by the workplace where the activities are performed than job satisfaction. In a similar vein, overall mental health is also likely to be influenced by one’s private life, to have a genetic component, etc.

Most studies on effects, rather than cross-sectional studies on associations, are experimental in nature, conducted under laboratory conditions. Such studies are necessarily limited to short-term effects. Field studies, with interventions in the daily working environment, are less common. Thatcher et al. (2020) shows that results from experimental studies are not always replicated in field studies with a longer period of post measurement. In other words, the ecological validity of experimental studies seems to be limited. Therefore it is important to conduct such field studies as well. This study was set up to do precisely that, including post-measurements over a longer period of time.

## 2. Method

### 2.1. Research locations

Nine organizations were found willing to participate in the study. This implied paying for the plants themselves, as well as for their installation and maintenance by a professional organization (at least during the study), the placement of sensors, and allowing employees to fill out questionnaires during work-time, as well as covering part of the research costs. In each organization, at least one workspace was selected as intervention office and at least one workspace as control office. In some organizations, multiple workspaces per condition were selected to increase the number of potentially participating employees. Selection criteria for both workspaces were:

- Employees spending a considerable part of their working hours in this workspace, to maximize the potential influence of the physical environment (flex desks should be located within the same workspace)
- Preferably open plan offices, as plants may have more effect(s) in such workspaces (e.g., with regard to privacy and noise annoyance)
- Preferably no green window views, as these might compensate of the absence of indoor plants (higher floors within the same building were preferred)
- Number of employees working in the (one or more) workspace(s) selected for a condition should be considerable, to be able to conduct a quantitative analysis
- Similarity in type of activity performed during working time in the selected workspaces, for maximum comparability
- No or only a few plants present in the workspaces before the start of the study, as removing plants (especially in the control workspace) is likely to reduce employees’ willingness to participate in the study.

Employees were not randomly assigned to a specific condition but worked in their usual office. Therefore, this field study has a quasi-experimental design.

### 2.2. Intervention

One of the goals of the project was to study the effect of potted plants on the indoor climate. This goal determined the shaping of the intervention. The type, size and number of plants was chosen so as to increase the relative humidity in the workspace by 15%-point, based on an extrapolation of their effect under laboratory conditions. This meant that a substantial number of plants was introduced. The precise number was based on the volume of the workspace and the humidifying capacity of the plants. Using a Chlorophytum (spider plant) with a pot size of 14 cm as reference plant, about 1 plant was calculated to be needed per 5 m^3^. An additional criterion was that the plants were low maintenance, so as to reduce costs. During the study, the plants were maintained by a professional organization and unhealthy plants were replaced, to ensure an optimal performance. Frequently used plants, next to Chlorophytum, were Spathiphyllum, Clusia, Kentia, Asplenium antiquum. The design of the intervention was made by the professional organization, also taking into account the desires of the organization and practical limitations and costs. Designers were asked to put the plants in the field of view of the office workers as much as possible. The level of participation of employees in the design process was left up to the organization.

### 2.3. Measurements

Measurements consisted of continuous sensor measurements and of several rounds of web-based questionnaires. The sensors measured four characteristics, starting at least a month before the introduction of the plants in the office: ambient temperature, relative humidity, CO_2_-level, concentration of volatile organic compounds (VOCs). More information on this part of the study can be found elsewhere (Hermans et al., 2022). The questionnaire included questions regarding all the variables in the conceptual model (see Figure 1), with exception of the objective characteristics: relative humidity (B.1), air quality (B.2), sound level (B.3), and the number of plants in one’s field of sight (B.4). The first two were covered by the sensor measurements. No objective sound measurements were conducted. Given the substantial number of plants that was introduced, the number of plants in sight was assumed to be larger after their introduction than beforehand. As for complaints about dry air (C.1), participants were asked whether during the preceding week the air had been too dry or not (yes/no). A similar format was used for noise annoyance (C.2): had they experienced too much noise or not. For thermal comfort (C.3), two of such questions were asked: experienced the temperature as being too high, and experienced the temperature as being too low. Answers were combined in a single thermal discomfort measure: too high and/or too low (1) or neither of both (0). For sense of privacy (C.4), participants were asked to what extent they agreed with the statement that their workspace offered them enough privacy (5-point scale: completely disagree—completely agree). A similar format was used for the visual appearance of the workspace (C.5): my workspace is attractive to look at.

Regarding the satisfaction with their workspace (D.1), participants were asked how satisfied they were overall with their workspace (5-point scale: very dissatisfied—very satisfied). Affect (D.2) was measured with a Dutch version of the job-related affective well-being scale developed by Warr (1990), as included in the Dutch Questionnaire “Vragenlijst Beleving en Beoordeling van de Arbeid” (VBBA, 2000-version; Van Veldhoven et al., 2002). Participants were asked to what extent each of twelve mood states, such as nervous or cheerful, did apply to them during the preceding week when they were at work (4-point scale: not at all—completely). The six positive mood states were combined in positive affect scale (D.2a) and the six negative ones in a negative affect scale (D.2b). The self-reported stress level (D.3) of the participant in the preceding week was measured with the seven items of the stress-subscale of the Depression Anxiety Stress Scale (Dutch-revised version of DASS-21; De Beurs et al., 2001). Answers had to be given on a 7-point scale (not at all/never—most certainly/mostly). The self-reported ability to concentrate (D.4) was measured with a scale that we developed ourselves and has been successfully used in a previous study (Hermans et al., 2019). This scale consists of six statements with participants having to indicate to what extent they agree with the statement (7-point scale: completely disagree—completely agree). Three statements were positively formulated and the other three negatively; the answers to the negative ones were recoded, making a higher score indicative of a higher ability to concentrate. For health-related complaints (D.5), we used a subset of a Dutch checklist for the indoor climate, employed by working conditions services. The six included complaints were: dry, itchy or irritated eyes, tired or strained eyes, sore or dry throat, headache, fatigue, drowsiness. For each complaint, it was asked (i) whether or not it applied to the participant in the preceding week (yes/no), and if so (ii) if the complaint got worse when at work. The six items were reduced to a single dichotomy: no complaint that got worse when at work (0) versus at least one complaint that got worse when at work (1).

The social climate in the workplace (E.1) was assessed with a scale that is another part of a frequently used questionnaire on how employees experience and assess different aspects of their job (VBBA, 2000-version). The scale focuses on relationships with colleagues and consists of nine items, such as: “are your colleagues friendly towards you?,” to be answered on a 7-point scale (never—always). Six of the nine items are positively formulated and three negatively. The answers to the three negatively formulated items are recoded, so that a higher score indicates a more positive social climate. Work pleasure (E.2) was assessed by the level of applicability with a single statement: “I generally enjoy going to work” (7-point scale: almost never—almost always). Self-assessed functioning (E.3) was measured by a single question: “How satisfied are you with your own functioning at work?” (7-point scale: very dissatisfied—very satisfied). Need for recovery (after work) was assessed with the Need-for-recovery scale of the VBBA-2000 questionnaire. The answers to the eleven items were coded in such a way that a higher score indicates a higher need for recovery.

Mental health (F.1) was assessed by the Dutch version of the Mental Health Inventory (MHI-5; Van Beljouw and Verhaak, 2010). For reporting in sick (F.2), participants were asked whether this had occurred in the preceding three months (yes/no), which is shorter than the interval between measurement rounds. Finally, job satisfaction (F.3) was measured with a single question: “How satisfied or dissatisfied are you altogether with your job?,” to be answered on a 7-point scale (very dissatisfied—very satisfied). When looking at the pre-measurement data, all multi-items scales with more than two answering options had a satisfactory internal consistency (Cronbach’s alpha of at least 0.75). The complete questionnaire is available in Dutch (Hermans et al., 2022).

During the pre-measurement, the above set of questions was preceded by some background questions, e.g., on gender and age. During the first post-measurement, the set of questions was followed up with some questions regarding the intervention, but only for participants in the intervention condition. In all cases, answer had to be given on a 7-point scale. The first question was whether the participant thought that generally speaking having plants in the workspace was a good idea (not at all—very good). The second question was whether the participant thought that this idea was implemented in a good manner (could have been much better—very good). The third question was whether the participant thought that the new plants contributed positively to the appearance of the workspace (not at all—very much so). The fourth question was whether the participant thought that the new plants contributed positively to his or her health and wellbeing (not at all—very much so).

Rounds of questionnaire-based measurements were scheduled once every 4 months, for a period of 2 years. Ideally, these measurements would have started in all organizations all at the same time, with the first, pre-measurement taking place in May/June 2019, the introduction of the plants 1 month later and the first post-measurement in September/October of that year. However, the time of enrollment differed between organizations, and therefore also the time of the first measurement. Organizations that joined the project later on were expected to participate in fewer rounds of measurements, due to the limited runtime of the project. Due to the COVID-19 pandemic, the five measurement rounds scheduled after February 2020 were cancelled. In the five organizations that did start with the pre-measurement in May/June 2019, two post-measurements were conducted. In two other organizations, only one post-measurement was conducted. Finally, in the last two organizations, entering the project even later, not a single post-measurement could be conducted. Furthermore, organization 2 was excluded from the effect analyses due to problems with the implementation of the intervention: part of the plants intended for the intervention workspace were placed in the control workspace. However, the pre-measurement data of all nine organizations were included in correlational analyses.

### 2.4. Participants and response

Participation of employees in the study, consisting of filling out questionnaires, was voluntary. Care was taken that the employer and manager could not identify who participated and who did not. However, employers and managers were asked to help and motivate their employees to participate. Overall, 594 of the 1,134 invited employees participated in the pre-measurement (52%), with participation levels differing substantially between the nine organizations. Keeping employees involved in the study proved to be difficult, even more so in the control conditions (see Table 1). For the within-subject analyses, employees need to have participated in several measurement rounds. Of the 594 participants in the pre-measurement, 288 employees also participated in the first post-measurement, of which 254 filled in both questionnaires completely. 146 Employees participated in both the pre-measurement and the second post-measurement, with 128 of them having filled in the questionnaires completely. Note that the substantial drop in numbers of participants is also due to a post-measurement round not having been conducted in some organizations, due to COVID-19.

**Table 1:** Number of employees in selected workspaces, by organization, condition and percentage participating by measurement round.

Organization	Condition	Number of employees	Participation in pre (%)	Participation in post 1 (%)	Participation in post 2 (%)
1	Intervention	213	41	X	X
	Control	127	43		
2	Intervention	42	83	54	51
	Control	63	70	42	26
3	Intervention	18	89	80	X
	Control	45	73	48	
4	Intervention	87	41	X	X
	Control	15	33		
5	Intervention	151	42	48	X
	Control	133	35	34	
6	Intervention	30	50	48	32
	Control	36	42	32	19
7	Intervention	47	74	49	41
	Control	26	77	52	58
8	Intervention	22	86	86	90
	Control	26	77	56	50
9	Intervention	27	93	92	92
	Control	26	92	92	65

X signifies that the measurement round did not take place in that organization, due to the COVID-19 pandemic (not all organizations entered the study at the same time).

Overall, in the intervention condition 54% of the employees was female. In the control condition this was 51%. There were substantial differences in this respect between organizations, also between the two conditions within the same organization. The average age in the intervention condition was 41 and 42 in the control condition. There were differences between organizations in the average age, but the difference between the two conditions within the same organization tended to be small, with an exceptional high 9 year difference for organization 4.

### 2.5. Analyses

To start with, bivariate correlations between the variables in the conceptual model were calculated, based on the pre-measurement data of all nine organizations. This was done to see if the hypothesized associations between the different outcome variables were indeed present, and if so, to what extent. Since it is a cross-sectional analysis, it gives no information on the causality of observed associations. Furthermore, this analysis does not provide information on the effect of the intervention on the outcome variables. For the analysis of the effect of the introduction of the plants in the workspace, the data from organization 2 were removed. In this organization, part of the plants intended for the intervention office had been placed in the control office instead, out of fairness considerations. The effect of the intervention was analyzed twice: once for all six organizations with at least one post-measurement, and once for only the four organizations with two post-measurements. In these analyses, the intervention effect takes the shape of an interaction between the condition (intervention vs. control) and the measurement (Pre vs. Post(s)). The hypotheses are that these difference-in-differences analyses will show more positive/less negative change in each of the outcome variables in the intervention condition than in the control condition. In the analyses with two post-measurements, two contrasts were specified: that between the two post-measurements and the pre-measurement (1), and that between the second and the first post-measurement (2). The first contrast pertains directly to the hypotheses. As for the second contrast, no hypotheses were formulated with regard to the trajectory of the effect of introducing plants after their introduction, making it an exploratory analysis. The design of these analyses was repeated measures linear mixed model for outcomes variables that could be considered continuous, and repeated measures generalized linear mixed model for dichotomous outcome variables, both with a random intercept for organization, a first-order autoregressive covariance structure for the repeated measures and a variance components structure for the random intercept. Analyses were conducted using SPSS (versions 25/28).

## 3. Results

### 3.1. Correlational results (based on pre-measurement data)

As a first check on the plausibility of the conceptual model, bivariate correlations were calculated between all outcome variables, ranging from C.1 to F.3, based on the data of the pre-measurement in all nine organizations. For mood (D.2), two subscales were used: one for positive affect (D.2a) and one for negative affect (D.2b).

Table 2 shows that many outcome variables are significantly related to each other, although not always very strongly so. In the case of a significant correlation, the sign of the correlation is always as expected. Within the C-segment of the model, seven of the 10 intercorrelations are significant, with only the one between noise annoyance (C.2) and privacy (C.4) above an absolute 0.30-threshold that we used to identify stronger associations: r = −0.33. Within the D-segment, all 15 intercorrelation are significant, with 7 of them above the 0.30-threshold. The strongest correlation is that between negative affect (D.2b) and stress (D3; r = 0.66). Within the E-segment, all six intercorrelations are significant as well, with five of them above the 0.30 threshold. The strongest correlation was that between the social climate (E.1) and work pleasure (E2; r = 0.50). Within the F-segment, also all three intercorrelations were significant, with only the one between mental health (F.1) and job satisfaction (F.3) being higher than 0.30: r = 0.46.

**Table 2:** Bivariate correlations between the outcome variables in the conceptual model.

	C.2	C.3	C.4	C.5	D.1	D.2a	D.2b	D.3	D.4	D.5	E.1	E.2	E.3	E.4	F.1	F.2	F.3
C.1 *	0.15	0.27	−0.16	−0.15	−0.18	−0.03	0.13	0.08	−0.09	**0.34**	−0.04	−0.05	−0.04	0.09	−0.09	0.01	−0.04
C.2 *	1	0.11	**−0.33**	−0.07	−0.24	−0.10	0.12	0.16	**−0.31**	0.13	−0.08	−0.15	−0.10	0.12	−0.04	0.04	−0.14
C.3 *		1	−0.06	−0.04	−0.10	−0.01	0.09	0.04	−0.06	0.26	−0.05	−0.09	−0.03	0.06	−0.09	0.07	−0.12
C.4			1	0.21	**0.31**	0.15	−0.13	−0.15	0.29	−0.11	0.14	0.14	0.11	−0.14	0.08	−0.06	0.16
C.5				1	**0.42**	0.18	−0.15	−0.15	0.19	−0.18	0.06	0.17	0.04	−0.14	0.13	−0.01	0.16
D.1					1	0.25	−0.21	−0.22	0.30	−0.23	0.17	0.22	0.07	−0.24	0.16	0.01	0.25
D.2a						1	**−0.56**	**−0.53**	**0.34**	−0.12	**0.40**	**0.53**	**0.35**	**−0.48**	**0.56**	−0.07	**0.46**
D.2b							1	**0.66**	**−0.40**	0.24	**−0.42**	**−0.49**	**−0.36**	**0.54**	**−0.63**	0.06	**−0.43**
D.3								1	**−0.51**	0.25	**−0.37**	**−0.45**	**−0.33**	**0.56**	**−0.51**	0.03	**−0.36**
D.4									1	−0.28	0.22	**0.36**	**0.34**	**−0.47**	**0.31**	−0.10	0.30
D.5										1	−0.14	−0.23	−0.14	0.26	−0.18	0.13	−0.15
E.1											1	**0.50**	**0.31**	−0.27	**0.39**	−0.09	**0.47**
E.2												1	**0.47**	**−0.37**	**0.53**	−0.09	**0.74**
E.3													1	**−0.39**	**0.41**	−0.09	**0.46**
E.4														1	**−0.55**	0.15	**−0.33**
F.1															1	−0.15	**0.46**
F.2 *																1	−0.11

Outcome variables: C.1—complaints of dry air, C.2—noise annoyance, C.3—thermal comfort, C.4—sense of privacy, C.5—attractiveness workspace, D.1—satisfaction with workspace, D.2a—positive mood, D.2b—negative mood, D.3—perceived stress, D.4—concentration, D.5—health-related complaints, E.1—social climate, E.2—work pleasure, E. 3—functioning, E.4—need for recovery, F.1—mental health, F.2—reporting sick, F.3—job satisfaction (see also Figure 1).

*Dichotomous variable.

Bold: significant at *p* < 0.05 and |r| > 0.30.

Grayed: not significant at *p* < 0.05 (all other values significant at *p* < 0.05).

Outcome variables in the C-segment of the model are 25 out of 30 times significantly correlated with those in the D-segment, of which four times about the 0.30-threshold. The strongest correlation is that between the visual attractiveness of the workspace (C.5) and the overall satisfaction with the workspace (D.1; r = 0.42). Outcome variables in the C-segment are 11 out of 20 times correlated with those in the E-section, but never above the 0.30-threshold. For the correlations with the F-segment, this is seven out of 15 times, but also never above the 0.30-threshold. Outcome variables in the D-segment are 23 out of 24 times correlated with those in the E-segment, and 15 times above the 0.30-threshold. The strongest correlation is that between stress (D3) and need for recovery after a workday (E4; r = 0.56). Outcome variables in the D-segment are 14 out of 18 times correlated with those in the F-segment, of which seven times above the 0.30-threshold. The strongest correlation is that between negative affect (D.2b) and mental health (F.1; r = −0.63). Finally, outcome variables in the E-segment are 12 out of 12 times correlated with those in the F-segment, of which eight times above the 0.30-threshold. The strongest correlation is that between work pleasure (E.2) and job satisfaction (F.3; r = 0.74).

### 3.2. Assessment of the intervention by employees

At the end of the first post-measurement, participants in the intervention conditions were asked four questions regarding this intervention. Since the intervention was organization-specific to some extent (choice of plants, positioning), the outcomes are presented by organization. Outcomes are limited to the organizations at which at least a first post-measurement took place. Given a scale maximum of seven, in all seven organization the idea of plants in the workspace was considered favorably. The way that the intervention was executed scored less high, especially in organization 7, but was in all organizations clearly above the scale midpoint of four, i.e., on the positive side of the scale. The contribution of the plants to the appearance of the workspace was rated quite positively, although somewhat less so in organizations 7 and 9. Finally, regarding whether the plant contributed to one’s own well-being, the answer were on average also quite positive. All in all, the intervention was well received in all seven organizations, with the employees also perceiving benefits for their own well-being (Table 3).

**Table 3:** Perceptions of employees regarding the intervention: average answers given on 7-point scales (1–7), with higher scores indicating more positive perceptions.

	Plants good idea	Idea well executed	Contributes to appearance	Contributes to own well-being
Organization 2 (*n* = 19)	6.3 (1.0)	5.9 (1.0)	6.5 (0.6)	5.6 (1.2)
Organization 3 (*n* = 15)	6.5 (0.6)	5.4 (1.2)	6.0 (1.0)	5.2 (0.9)
Organization 5 (*n* = 56)	6.8 (0.5)	5.7 (1.3)	6.3 (1.1)	5.8 (1.3)
Organization 6 (*n* = 14)	6.7 (0.5)	5.5 (1.5)	6.0 (1.1)	6.1 (1.2)
Organization 7 (*n* = 20)	6.1 (1.3)	4.8 (1.8)	5.3 (1.8)	5.3 (1.5)
Organization 8 (*n* = 17)	6.8 (0.8)	5.5 (0.8)	6.2 (0.8)	5.2 (0.9)
Organization 9 (*n* = 24)	6.0 (1.1)	5.0 (1.7)	5.4 (1.4)	5.1 (1.3)

In organizations 1 and 4 no post-measurements were conducted (due to COVID-19). Questions asked in intervention conditions only. Standard deviation in parentheses.

### 3.3. Effect analyses

Since in organizations 1 and 4 no post-measurement took place (due to COVID-19), and in organization 2 the intervention was not correctly implemented, only six organizations (3, 5, 6–9) were included in the mixed model analyses looking at the development in the intervention office between the pre-measurement and the first post-measurement (*n* = 390). These analyses show significant intervention effects for only three of the eighteen outcome variables (see Table 4).

**Table 4:** Overview of mixed model effect analyses for all outcome variables.

Outcome variable	Pre vs. post 1 *F*-value (df1, df2)	Pre vs. post 1 & 2 *F*-value (df1, df2)
C.1 Air too dry^b^	**7.76 (1, 601)** ^**^	**4.98 (2, 429)** ^**^
C.2 Too noisy^b^	0.04 (1, 601)	2.46 (2, 429)
C.3 Thermal discomfort^b^	1.47 (1, 601)	0.19 (2, 429)
C.4 Privacy^a^	**5.41 (1, 288)** ^*^	**6.46 (2, 288)** ^**^
C.5 Appearance workspace^a^	**21.13 (1, 300)** ^***^	**9.41 (1, 290)** ^***^
D.1 Satisfaction with workspace^a^	2.70 (1, 308)	**3.46 (2, 269)** ^*^
D.2a Positive affect^a^	1.38 (1, 288)	0.54 (2, 273)
D.2b Negative affect^a^	1.89 (1, 287)	0.25 (2, 275)
D.3 Stress^a^	0.24 (1, 260)	0.00 (2, 247)
D.4 Ability to concentrate^a^	0.86 (1, 264)	0.75 (2, 257)
D.5 Health-related complaints^b^	3.20 (1, 594)	**3.25 (2, 427)** ^*^
E.1 Social climate^a^	0.04 (1, 262)	2.14 (2, 255)
E.2 Work pleasure^a^	0.55 (1, 245)	0.08 (2, 245)
E.3 Own functioning^a^	0.28 (1, 308)	0.23 (2, 286)
E.4 Need for recovery^a^	0.86 (1, 256)	0.21 (2, 248)
F.1 Mental health^a^	0.13 (1, 268)	1.47 (2, 257)
F.2 Reporting sick^b^	0.37 (1, 565)	2.67 (2, 407)
F.3 Job satisfaction^a^	0.13 (1, 258)	0.17 (2, 259)

*,**,*** Significant at *p* < 0.05, 0.01, and 0.001, respectively. In parentheses, df1 and df2, refer to the degrees of freedom for nominator and denominator for the F-test, respectively. Bold, significant at least *p* < 0.05.

a Linear mixed model.

b Generalized mixed linear model.

The estimated (marginal) means show that after the intervention fewer people in the intervention condition complained about the air being too dry (23%) than before (45%), whereas in the control condition the percentage remained almost the same: before—38%, after—37%. As for perceived privacy, the intervention condition shows an increase (M_pre_ = 2.63; M_post1_ = 3.06), whereas this is not/less the case in the control condition (M_pre_ = 2.67; M_post1_ = 2.80). A similar pattern is observed for the appearance of the workspace. Its attractiveness increased in the intervention condition (M_pre_ = 2.90; M_post1_ = 3.53), whereas it remained virtually the same in the control condition (M_pre_ = 2.99; M_post1_ = 3.04).

**Figure 2:**
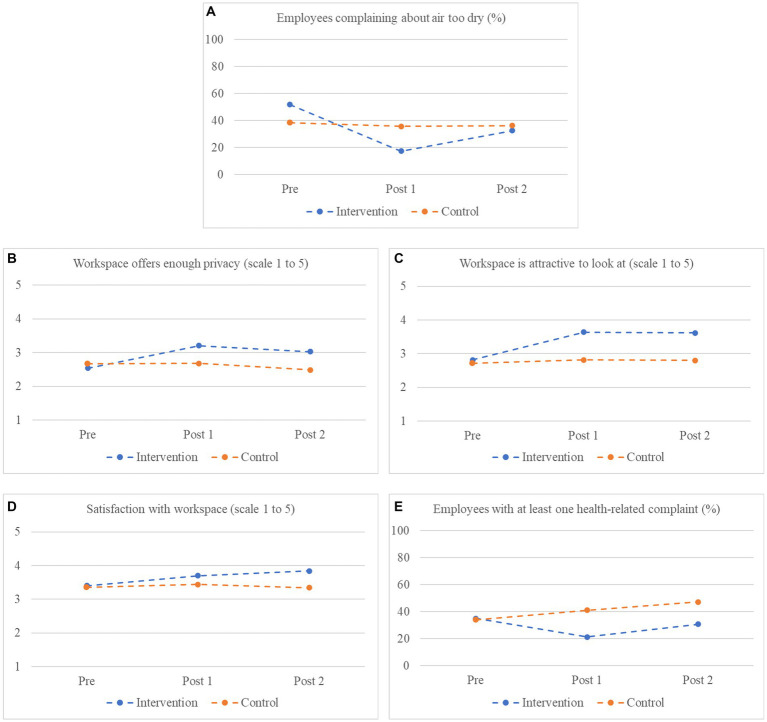
**(A–E)** Pre-and post-measurement scores (percentage or mean) for the five outcome variables showing significant effects in the mixed model analyses with both post-measurements included, based on organizations 6–9.

Due to missing second post-measurements, only four organizations (6–9) were included in the mixed model analyses looking at the development in the intervention office between the pre-measurement and the two post-measurements taken together (*n* = 202). These analyses showed significant effects for five outcome variables (see Table 4). In all five cases, only the contrast comparing the pre-measurement with the two post-measurements was significant; no significant differences between the two post-measurements were observed. The first outcome variable showing a significant effect of the intervention is the percentage of participants complaining about the air in the workplace being too dry (see Figure 2A). Whereas during the pre-measurement this percentage was higher in the intervention condition, at the first post-measurement it percentage dropped below that in the control condition, which remained more or less the same as during the pre-measurement. At the second post-measurement the percentage increased somewhat, and become more or less the same as that in the control condition, which was still about the same as before. Thus, there is a clear initial effect, that may have been helped by the complaints originally being more common in the intervention condition, indicating that there was more room for improvement. For perceived privacy and the visual appearance, both conditions started at the same level at the pre-measurement, but after the introduction of the plants both the perceived privacy and the visual appearance of the workspace were rated more positively in the intervention condition. This difference remained the same at the second post-measurement (see Figures 2B,C). Thus, the effects on perceived privacy and visual appearance, and to a lesser extent that on the percentage of participants complaining about the air being too dry, persisted for at least 4 months after the intervention.

In the analyses including two post-measurements also two new effects emerged. The first was the overall satisfaction with the workspace increasing in the intervention condition. The pattern more or less suggests that the effect already started at the first post-measurement, but increased over time (see Figure 2D). The other effect that emerged involved the percentage of participants with at least one health-related complaint that got worse when at work. This percentage was the same at the start, but dropped in the intervention condition after the introduction of the plants, whereas there was a slight increase in the control condition. Between the first and second post-measurement there was slight increase in both conditions, but the difference between the two conditions remained about the same (see Figure 2E). It should be noted that, in the analyses including both post-measurements, only those employees that participated in all three measurements are included. Visual inspection of the means for the first three effects suggests that the effect of the plants at the first post-measurement was larger for the subset of participants that also completed the second post-measurement than for the subset of participants that did not.

### 3.4. Attrition, representativeness, and moderation

In studies taking place over a longer period of time, with multiple rounds of measurement, attrition can constitute a problem, especially if the dropout of participants is selective. As a check, employees of organizations that participated in both measurement rounds but themselves only participated in the pre-measurement (*n* = 123) were compared to those that participated in the first post-measurement as well (*n* = 288). For the 18 outcome measures, this was done by either cross-tabulation (for dichotomous outcome variables) or by analysis of variance (for interval outcome variables), taking also condition into account (but not organization). At the time of the pre-measurement, both groups did not differ on the outcome variables, with one exception. A higher percentage of the employees that only participated in the pre-measurement had experienced too much noise than those that also participated in the first post-measurement: 74% vs. 61% (Chi^2^ (1) = 5.58; *p* < 0.05). However, this occurred to the same extent in the intervention condition (77% vs. 63%) and in the control condition (71% vs. 59%). Also, there is no effect of the intervention on this outcome variable to begin with, making the question of a spurious effect due to attrition less relevant.

The two groups were also compared with regard to their composition in terms of gender and age. With regard to gender, in the intervention condition there was a selective dropout of males (Chi^2^ (1) = 5.56; *p* < 0.05). Of the employees that only participated in the pre-measurement 61% was male, whereas of those that also participated in the first post-measurement this was 42%. In the control condition the difference between the two groups was not significant (48% vs. 51%). To see to what extent this selective dropout might have affected the outcomes regarding the effect of the intervention, the mixed-model analyses were repeated for the outcome variables that previously showed a significant effect of the introduction of the plants. Moderation of the effect of the intervention by gender is indicated by a significant three-way interaction effect: condition by measurement round [pre vs. post(s)] by gender. For the three outcomes variables showing an effect in the analyses with only the first post-measurement compared to the pre-measurement, as well as for the five outcome variables showing an effect in the analyses with both post-measurement compared to the pre-measurement, no significant three-way interaction with gender was observed. Furthermore, in all eight analyses the original (two-way interaction) effect remained significant.

## 4. Discussion

Introducing a substantial number of plants in the office affected a few of the 18 outcome variables that were distinguished in our conceptual model. The effects concerned rather proximal outcome variables, i.e., variables that are closely positioned to the introduction of plants in the model, such complaints about the air being too dry and the attractiveness of the appearance of the workspace. This is in line with what one would expect, and also consistent with the rather weak (cross-sectional) correlations of these proximal outcome variables with the more distal ones, based on the pre-measurement data. On the other hand, the effects that were observed were not short-lived. Also at the second post-measurement, at least 4 months after the introduction of the plants, three of the five effects observed in the analyses that included both post-measurements are about of the same size as at the first post-measurement. Moreover, in these analyses effects for more outcome variables were observed than in the analyses in which only the first post-measurement was included. It should be noted, however, that in the analyses including both post-measurements fewer organizations (4) were involved than in the analyses that only looked at the difference between the first post-measurement and the pre-measurement (6), due to the COVID-19 pandemic. Visual inspection suggests that, in the four organizations involved in both analyses, effects at the time of the first post-measurement were somewhat stronger than in the two organizations only involved in the analyses with one post-measurement. In terms of the way participants in the intervention condition of the different organizations assessed the introduction of the plants, the invention does not appear to have been less successful in the latter two organizations. However, organizations with only one post-measurements enrolled later in the research project. As a consequence, the timing of the different measurements differed from those organizations with two post-measurement. Therefore, the season in which a particular measurement took place may have influenced the effect of the plants.

As we mentioned in our introduction, Thatcher et al. (2020) showed that effects observed in experiments under laboratory conditions cannot always be replicated in intervention studies conducted in real-life settings. We want to point out that despite the participants being quite positive about the intervention, and expecting that these would affect their own well-being in a positive way, we did not observe effects on mood when at work, or on mental well-being in general. Despite methodological issues regarding this comparison, we are inclined to conclude that there is a difference in how employees expect the introduction of plants in their work space to affect their well-being and the actual change in their well-being, with the expected impact being larger than the actual impact. A similar tentative conclusion may be drawn with regard to the comparison of intervention studies and cross-sectional studies. In their systematic review, Zhao et al. (2022) identified six cross-sectional studies looking into the association between the presence of indoor plants and mental well-being. They concluded that in general favorable effects of indoor plants are supported. However, such an effect on this more distal outcome variable was not observed in our intervention study.

In the present study, the intervention was optimized for improving relative humidity, and not for improving the appearance of the workspace. The latter might have led to a different selection on plants, e.g., more ornamental, more fast growing, more colorful, more flowering. Although there are studies looking into the effect of the type of plant (see, e.g., Elsadek and Liu, 2021; Li et al., 2021), evidence regarding the relevance of such characteristics is quite limited. In their systematic review, Zhao et al. (2022) ask for more research on what they call the quality metrics, besides on the quantity metrics of indoor plants. As for the quantity of indoor plants, perhaps not that many are needed, or even preferable. Pérez-Urrestarazu et al. (2021) conclude that in a domestic context a few indoor plants placed in strategic positions were preferred to a high number of plants. Toyoda et al. (2020) observed a stress-reducing effect of even having only a single small plant (of choice) on one’s desk, although this was in combination with the instruction to take a 3-min rest when feeling fatigued. They were also instructed to do so during the pre-intervention phase of the experiment, but in the post-intervention phase of the experiment, employees were assumed to gaze intentionally at their plant during such rests. We would like to point out that also research on the psychological effect of the number of plants is limited, especially when it comes to long-term effects in field settings. Han et al. (2022) conducted a systematic review of the effects of plants on human functions (note: not on well-being) and identified 42 eligible studies, of which most were laboratory experiments, with short exposures to the plants. Of the 11 included field (quasi-)experimental studies in this review, only one compared different numbers of plants (Larsen et al., 1998), with a limited exposure time: 15–20 min.

### 4.1. Strengths and limitations

This study is one of the few that investigated the effects of plants in the daily workspace setting over a longer period of time, with an exposure to the plants of at least up to 4 months. Moreover, it did so in multiple organizations, involving a substantial number of employees. Whereas the intervention being replicated in several organizations is definitely a strength of the study, the limited participation of the employees in the workspaces involved in the study is somewhat of a limitation with regard to the generalizability of the outcomes. The same is true for the attrition occurring during the study, with fewer participants in later measurement rounds. However, we were able to show that differences in attrition by gender are unlikely to have affected the outcomes substantially, since gender did not moderate the observed effects.

In this study, the number and type of plants that were introduced were mainly selected based on their ability to increased the relative humidity in the workplace by a certain amount (with associated air quality benefits). A secondary criterium was that the plants were likely to survive in the office setting at low levels of maintenance (during the study provided by professionals). The plants were not chosen because of how attractive they were to the employees. This implies that selecting plants based on their attractiveness might increase their impact. It is also unclear whether the same impact could have been attained with fewer plants, i.e., at lower costs to the organization. Future research will have to show if this actually the case.

### 4.2. Final conclusions

Since the onset of the COVID-19 pandemic, many office workers started working from home. Post-COVID-19, many of them like to continue doing so, at least for part of their working hours. For employers that wish their employees to spend more time at the office, one way to achieve this might be to make the office environment more attractive to their employees. The present study shows that introducing plants in the office can help to do so.

## Data availability statement

The datasets presented in this study can be found in online repositories. The names of the repository/repositories and accession number(s) can be found at: Dans Easy https://doi.org/10.17026/dans-z72-7w4z

## Ethics statement

Ethical review and approval was not required for the study on human participants in accordance with the local legislation and institutional requirements. The patients/participants provided their written informed consent to participate in this study.

## Author contributions

SV designed the study, performed analyses, and drafted and revised the text. FL conducted data preparation, also conducted analyses, and commented on drafts. TH acquired the funds for the study, recruited the participating organizations, and also commented on drafts. All authors contributed to the article and approved the submitted version.

## Funding

This study was supported by the Topsector Horticulture and Starting Materials (TU-18047).

## Conflict of interest

The authors declare that the research was conducted in the absence of any commercial or financial relationships that could be construed as a potential conflict of interest.

## Publisher’s note

All claims expressed in this article are solely those of the authors and do not necessarily represent those of their affiliated organizations, or those of the publisher, the editors and the reviewers. Any product that may be evaluated in this article, or claim that may be made by its manufacturer, is not guaranteed or endorsed by the publisher.
